# Effects of low-frequency rTMS combined with antidepressants on depression in patients with post-stroke depression: a systematic review and meta-analysis

**DOI:** 10.3389/fneur.2023.1168333

**Published:** 2023-05-19

**Authors:** Juanhong Pan, Hongpeng Li, Yongshen Wang, Li Lu, Ying Wang, Tianyu Zhao, Di Zhang, Song Jin

**Affiliations:** ^1^School of Health Preservation and Rehabilitation, Chengdu University of Traditional Chinese Medicine, Chengdu, Sichuan, China; ^2^School of Medical and Life Sciences, Chengdu University of Traditional Chinese Medicine, Chengdu, Sichuan, China; ^3^Rehabilitation Department, Hospital of Chengdu University of Traditional Chinese Medicine, Chengdu, Sichuan, China

**Keywords:** low-rTMS, antidepressants, PSD, depressive state, IL-6 and TNF-α levels, systematic review, meta-analysis

## Abstract

**Objective:**

To evaluate the effect of low-frequency (≤1 Hz) repetitive transcranial magnetic stimulation (low-frequency rTMS) combined with antidepressants on depression and the levels of inflammatory factors IL-6 and TNF-α in patients with post-stroke depression (PSD).

**Design:**

PubMed, Embase, Web of Science, Cochrane Library (CBM), China National Knowledge Infrastructure, Technology Periodical Database, and Wanfang Database were searched until October 2022 for randomized controlled trials.

**Participants:**

Patients with post-stroke depression (PSD) participated in the study.

**Results:**

A total of 16 randomized controlled trials (RCTs) involving 1,463 patients with PSD were included. According to the Physiotherapy Evidence Database (PEDro) quality assessment, three studies received high quality (eight scores) and 13 RCTs received moderate quality (six scores) results. The meta-analysis showed that low-rTMS combined with an antidepressant significantly reduced the Hamilton Depression Scale (HAMD) score and the National Institutes of Health Stroke Scale (NIHSS) score, reduced IL-6 and TNF-α levels, and improved the MMSE score in PSD compared to an antidepressant alone.

**Conclusion:**

The results of this meta-analysis evidenced the efficacy and safety of low-rTMS combined with antidepressants in the treatment of depression in PSD patients. The combined therapy could reduce The depression state and the levels of IL-6 and TNF-α, and enhance the cognitive function of patients. In addition, low-rTMS had fewer adverse effects, proving safety. However, there are shortcomings, such as a lack of long-term follow-up, different intervention sites of low-rTMS, and different intervention frequencies (0.5 or 1 Hz). Thus, in the future, RCTs with a larger sample size and longer-term observation are required to verify the efectiveness of low-rTMS combined therapy on PSD. Meantime, a new meta-analysis could be analysized, which intervention sites and frequency are more effective in treating PSD.

**Systematic review registration:**

https://www.crd.york.ac.uk/prospero/, identifier: CRD42022376845.

## Introduction

According to current reports, stroke is the second leading cause of death and the third leading cause of disability globally (1). The estimated prevalence, incidence, and mortality rate of stroke in China in 2020 were 2.6%, 505.2 per 100,000 persons per year, and 343.4 per 100,000 persons per year, respectively (2). Post-stroke depression (PSD) is one of the common complications after stroke. The incidence of PSD in China is at a high level, ranging from 20 to 70% (3–5), including 33% mild depression, 20% moderate depression, and 4% severe depression (6), and all-cause mortality in PSD patients increased by 59% (7). The main clinical manifestations of PSD are the experience of loss of interest, depression, sleep disorders, and anhedonia after stroke, which could accompany physical discomforts, such as pain and fatigue (8, 9). These not only lead to the aggravation of physical symptoms of stroke patients but also increase the burden on patients and their families, negatively impact their rehabilitation, and seriously reduce their quality of life.

The pathogenesis of PSD needs to be clarified, mainly including neurobiological mechanisms and social-psychological mechanisms. In studies of neurobiological mechanisms, some scholars have shown that the level of BDNF correlated with the incidence of PSD (10). At the same time, it is moderately positively correlated with the inflammatory factor tumor necrosis factor-α (TNF-α) in serum and highly positively correlated with the level of interleukin IL-6 (11).

There are two types of clinical treatment methods for PSD: drug and non-drug. Drug therapy is the primary treatment method, including selective serotonin reuptake inhibitors (SSRIs), SNRIs (serotonin-norepinephrine reuptake inhibitors), Na SSAs (non-noradrenergic and specific serotonergic antidepressants), and TCA (tricyclic antidepressant). However, the drug treatment has many adverse reactions, such as nausea, constipation, dizziness, insomnia, arrhythmia (12, 13), and even non-response to drugs (14). Therefore, we must find a better therapy to achieve a better therapeutic effect while reducing adverse reactions.

Repetitive transcranial magnetic stimulation (rTMS), a non-invasive brain neuro-modulation treatment method, can stimulate specific brain parts with specific frequencies to regulate the degree of nerve excitation and cortical function. It is the only brain stimulation treatment approved by the US Food and Drug Administration (FDA) as a non-invasive treatment for depression (15). In 2022, “Shanghai Expert Consensus on the Clinical Application and Operation Specification of Repetitive Transcranial Magnetic Stimulation” (16) also pointed out that rTMS were effective and safe in treating stroke. Many clinical studies have shown that rTMS combined with other therapies may be more effective than simple drug treatment (17, 18) or non-drug treatment (19, 20) in treating PSD.

Currently, many clinical studies were conducted on low-frequency rTMS combined with antidepressant therapy in treating PSD, but only a few systematic reviews exist (21, 22). Among them, Liang et al. (21) analyzed rTMS and transcranial electrical stimulation (TES) as non-invasive stimulation. However, only six studies analyzed low-rTMS combined antidepressants; the latest was only in 2018. Liang et al. (22) conducted a previous meta-analysis in 2018. Many of new literature has been published since 2018 (23–27). Some studies by some scholars have also shown that the appearance of PSD is related to the increase in IL-6 and TNF-α levels of the inflammatory factors IL-6 and TNF-α levels (28). However, these two relevant meta-analyses did not analyze the outcome indicators.

This meta-analysis aimed to update the efficacy and safety of low-rTMS (≤1 HZ) combined with antidepressant therapy in the treatment of PSD and to analyze the results of IL-6 and TNF-αlevels to provide a more reliable basis for the development of subsequent clinical research.

## Materials and methods

### Search strategy

**Figure 1:**
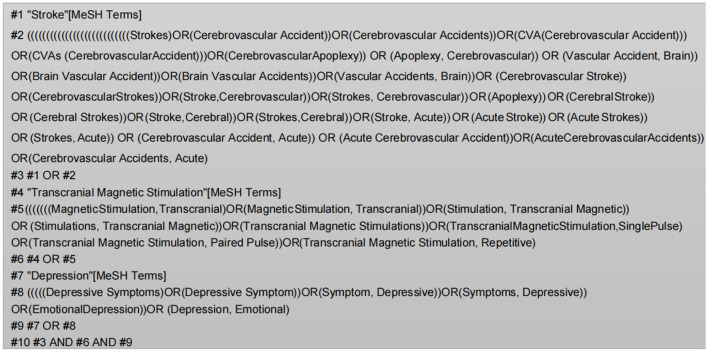
PubMed search history.

Two investigators (LL and JHP) conducted the study according to the search strategy for randomized controlled trials (RCTs) developed by the Cochrane Collaboration. We searched for seven major databases, PubMed, Embase, Web of Science, Cochrane Library (CBM), China's National Knowledge Infrastructure (CNKI), Technology Periodical Database (VIP), and Wanfang Database. The search time was from establishing the database to 10 October 2022. Chinese search keywords included “#1 stroke/ cerebrovascular accident/ cerebral hemorrhage/ cerebral ischemia/ cerebral thrombosis/ cerebral hemorrhage/ hemiplegia”; #2 “transcranial magnetic stimulation/ magnetic stimulation/ repetitive transcranial magnetic stimulation/ TMS/ rTMS”; #3 “depression/ depressive state.” English search was conducted using subject terms and free words (Figure 1), including “stroke,” “transcranial Magnetic Stimulation,” and “depression.” The language was limited to Chinese and English, and the study type was only required to be a randomized controlled trial (RCT).

### Inclusion criteria

After a review of the literature, we determined the eligibility criteria. This review uses the PICOS framework (population, intervention and research, comparison, and conclusion).

(1) People: The patients conforming to the Fourth National Cerebrovascular Disease Conference set criteria for diagnosing cerebral apoplexy and the International Classification of Diseases, 10th edition (ICD-10). These criteria regardless of gender differences, age, country, time, and race. (2) Intervention: Low-frequency repetitive transcranial magnetic stimulation (≤1 Hz) combined with antidepressants (such as duloxetine, mirtazapine, and fluoxetine) administered to patients, and both groups given essential treatment (such as nerve nutrition and vasodilatation) and rehabilitation training (passive movement, muscle strength training, and activities of daily living training). (3) Comparison: Antidepressants as a control intervention. (4) Outcome: At least one outcome index, namely, Hamilton Depression Scale (HAMD), interleukin-6 (IL-6), tumor necrosis factor-α (TNF-α), National Institutes of Health Stroke Scale (NIHSS), and overall efficacy. (5) Study: Randomized controlled trial (RCT) published in English or Chinese.

### Data extraction and management

Two researchers (JHP and HPL) independently searched and browsed seven databases according to the search strategy, imported the obtained literature into the Endnote X9 literature manager, selected the studies, and collected the data independently according to the inclusion and exclusion criteria. The extracted content included general information (name of the author, year of publication, and age of the participants) and study characteristics (experimental group and control intervention method, sample size, stimulation site, stimulation frequency, and duration). Any disagreements were negotiated and discussed with a third investigator (YSW) and finally reached a consensus.

### Quality assessment

The Physiotherapy Evidence Database (PEDro) was used to assess the quality of the literature. The PEDro scale uses 11 criteria, each rated as “yes” or “no,” and one point is awarded for each response. The first item is not included in the PEDro scoring, and the total score is 10 points. The total PEDro score of ≥7 points is of high quality, 5–6 points are of moderate quality, and ≤4 points are of low quality (39). The scores were given independently by two researchers (JHP and YW); if the results were inconsistent, they discussed them with a third researcher (YSW).

The two reviewers (LL and YW) also completed the risk of bias. The evaluation was carried out according to the Cochrane Handbook for Systematic Reviews of Interventions version 5.3. Items included the following: (1) random sequence generation (selection bias), (2) allocation concealment (selection bias), (3) blinding of participants and personnel (performance bias), (4) blinding of outcome assessment (detection bias), (5) incomplete outcome data (attrition bias), (6) selective reporting (reporting bias), and (7) other bias. The quality of the included studies was classified as low/unclear/high risk of bias (low risk of bias was “yes,” high risk of bias was “no,” otherwise was “unclear”).

### Statistical analysis

We used StataMP 17. for meta-analysis. Weighted mean difference (WMD) was used for continuous variables, while odd ratio (OR) was used as the effective statistics for dichotomous variables, and 95% confidence intervals (95% CI) were calculated for all data. The heterogeneity of the treatment effect was tested by calculating the *I*^2^ index. When *p* > 0.05 or *I*^2^ < 50%, it was considered low heterogeneity, and the fixed effect model was used for meta-analysis. When *p* < 0.05 or *I*^2^ > 50% was considered as high heterogeneity, meta-analysis was performed using a random-effects model, and sensitivity analysis was performed to identify the source of heterogeneity. Egger's test analyzed publication bias.

## Results

### Selection of the results

**Figure 2:**
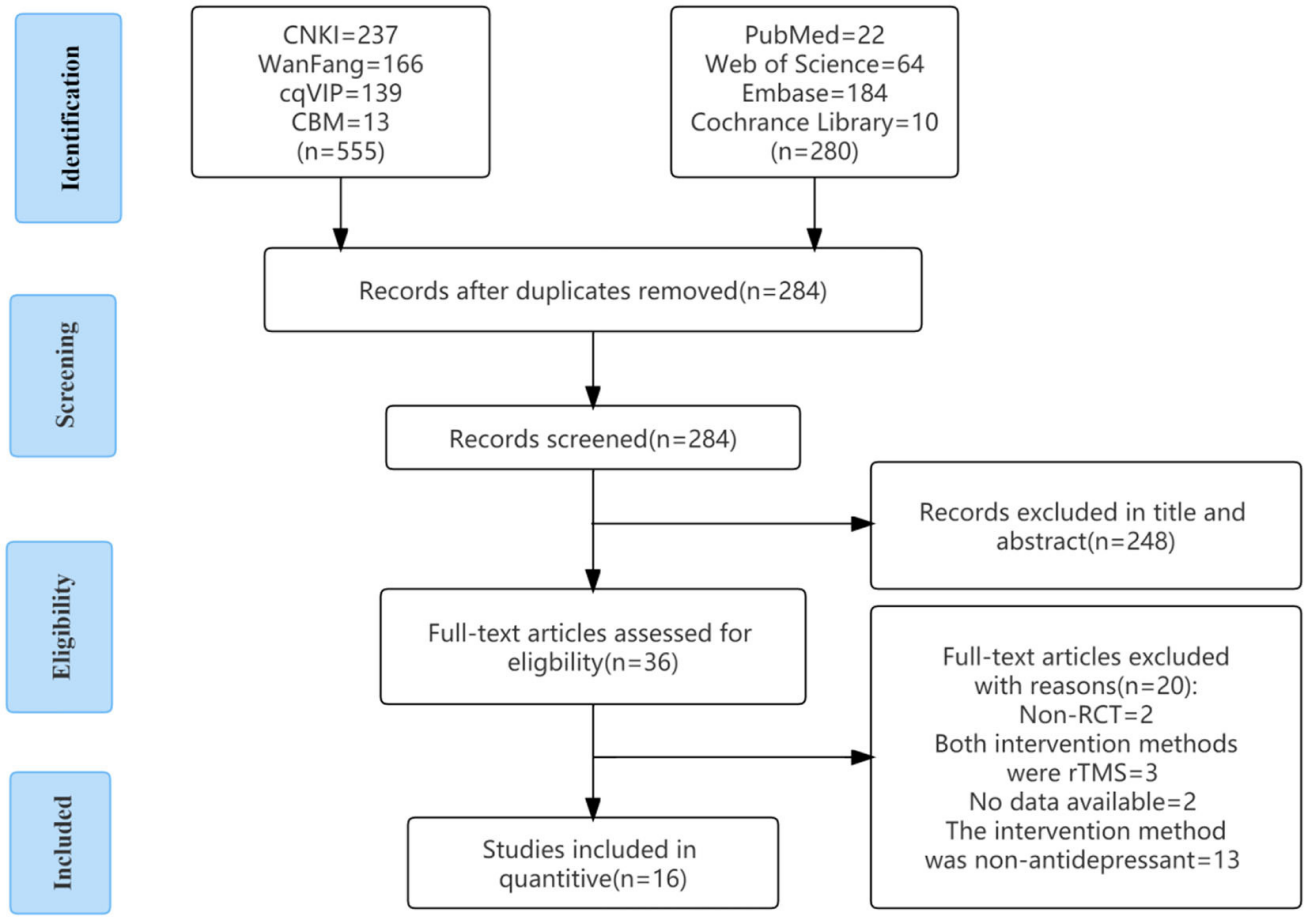
Flow chart of article selection. CBM, China Biology Medicine; CNKI, China National Knowledge Infrastructure; RCT, randomized controlled trial; VIP, Technology Periodical Database.

As of October 2022, we retrieved 835 pieces of literature according to the search strategy and excluded 551 duplicate literature. After reading the title and abstract, 248 articles were excluded. After reading the complete text, there were two randomized controlled trials, three studies with rTMS, two with no data extraction, and 13 with non-antidepressants. A total of 16 articles were included. The detailed screening procedure is shown in Figure 2.

### Study characteristics

The 16 (2, 23–27, 29–38) included articles were all randomized controlled trials on the effect of low-rTMS combined with antidepressants on PSD patients. A total of 1463 patients with PSD. Age was 18 years and older, a frequency of 5–7 days/week, and a course of treatment of 10 days to 12 weeks. The intervention group used rTMS combined with an antidepressant, and the control group's method was an antidepressant. The primary outcomes included the HAMD score and the overall response rate. The secondary outcomes included IL-6, TNF-α, NIHSS score, and MMSE score. Table 1 shows the detailed characteristics. There were no significant differences in the baseline data between the two groups.

**Table 1:** Characteristics of included studies.

**Study**	**Patients**	**Treatment group**						**Control group**					**Times**	**Outcomes**	**Positive/ negative**
		**Age (year)**	**Sample size**	**Intervention**	**Dose**	**Parts**	**Frequency**	**Age (year)**	**Sample size**	**Intervention**	**Dose**	**Frequency**			
Leyu and Lijun (29)	PSD	57.7 ± 4.3	32	rTMS + SSRI (fluoxetine)	0.5 Hz, 60% MT, 30 pulses/sequence, 1 sequence/day	Bilateral frontal lobe	1/day	58.3 ± 4.3	32	SSRI (fluoxetine)	20 mg/day	1/day	10 days	A + F	+
Guobing (26)	PSD	60.57 ± 5.38	36	rTMS + fluoxetine	0.5 Hz, 60% MT, 2.0 T	Bilateral frontal lobe	1/day	60.49 ± 5.31	36	fluoxetine	20 mg/day	1/day	4 weeks	A	+
Tian and Jia (30)	PSD	56.3 ± 7.1	63	rTMS + fluoxetine	0.5 Hz, 60% MT, 30 pulses/sequence, 1 sequence/day	Bilateral frontal lobe	1/day	56.0 ± 7.1	63	fluoxetine	20 mg/day	1/day	12 weeks	A + E	+
Xiaoyan (31)	PSD	61.43 ± 8.74	45	rTMS + duloxetine	1 Hz, 100% MT, 30 pulses/sequence, 10 sequence/day	Bilateral frontal lobe	1/day	64.78 ± 7.23	45	Duloxetine	60 mg/day	1/day	4 weeks	A + F	+
Jia et al. (32)	PSD	64.62 ± 11.45	50	rTMS + Flupentixol, melitracen tablets	1 Hz, 90% MT, 30 pulses/sequence, 1 sequence/day	Bilateral frontal lobe	1/day	66.50 ± 11.09	50	Flupentixol, melitracen tablets	10.5 mg/day	1/day	2 weeks	A	+
Jie (23)	PSD	57.66 ± 3.41	51	rTMS + fluoxetine	0.5 Hz, 80% MT, 30 pulses/sequence, 1 sequence/day	Right frontal lobe	1/day	58.27 ± 3.53	51	Fluoxetine	20 mg/day	1/day	4 weeks	A	+
Li et al. (33)	PSD	64.8 ± 5.4	26	rTMS + mirtazapine	1 Hz, 90% MT	Right frontal lobe	1/day	65.2 ± 4.8	27	Mirtazapine	15 mg/day−30 mg/day	1/day	4 weeks	A + D + F	+
Fan (34)	PSD	58.65 ± 6.01	20	rTMS + fluoxetine	1 Hz, 80% MT 800 pulses/day	Right frontal lobe	1/day	56.70 ± 5.95	20	Fluoxetine	20 mg/day	1/day	4 weeks	A + D	+
Niu et al. (24)	PSD	62.4 ± 4.3	50	rTMS + Paroxetine hydrochloride tablets	1 Hz, 90% MT, 1,500 pulses/day	Right frontal lobe	1/day	61.5 ± 4.1	50	Paroxetine hydrochloride tablets	20 mg−40 mg/day	1/day	8 weeks	A + B + C + D	+
Wang et al. (25)	PSD	68.52 ± 5.71	76	rTMS + paroxetine hydrochloride tablets	1 Hz, 800 pluses/day	Right frontal lobe	1/day, 5 days/week	68.39 ± 5.02	76	Paroxetine hydrochloride tablets	20 mg−40 mg/day	1/day	8 weeks	A + B + C + F	+
Tan et al. (35)	PSD	59.2 ± 4.2	26	rTMS + Escitalopram	1 Hz, 80% MT	Right frontal lobe	1/day, 5 days/week	57.4 ± 3.9	26	Escitalopram	10 mg/day−20 mg/day	1/day	6 weeks	A	+
Yunnan et at. (36)	PSD	55.4 ± 9.5	56	rTMS + fluoxetine	0.5 Hz, 30/sequence, 1 sequence/day	Left frontal lobe	1/day, 5 days/week	55.4 ± 9.5	56	fluoxetine	20 mg/day	1/day	8 weeks	A + E	+
Wang et al. (27)	PSD	64.98 ± 4.86	40	rTMS + Escitalopram	1 Hz, 90% MT, 50 pulses/sequence	Left frontal lobe	1/day, 5 days/week	65.27 ± 4.71	40	Escitalopram	5 mg/day−20 mg/day	1/day	4 weeks	A + B + C + F	+
Xiaoru et al. (37)	PSD	55.6 ± 5.8	60	rTMS + fluoxetine	1 Hz, 80% MT, 50 pulses/sequence, 30 sequences/day	Left frontal lobe	1/day, 5 days/week	55.8 ± 5.5	60	fluoxetine	20 mg/day	1/day	8 weeks	A + E + F	+
Kejiao (38)	PSD	54.77 ± 9.80	64	rTMS + fluoxetine	1 Hz, 80% MT, 50 pulses/sequence, 30 sequences/day	Left frontal lobe	1/day, 5 days/week	55.36 ± 10.90	64	fluoxetine	20 mg/day	1/day	8 weeks	A + E	+
Liu et al. (2)	PSD	55.61 ± 6.84	35	rTMS + paroxetine hydrochloride tablets	1 Hz, 70% MT, 30 s/sequence, 1 sequences/day	bilateral dorolateral	1/day, 5 days/week	50.20 ± 6.28	35	Paxil hydrochloride tablet	20 mg/day	1/day	8 weeks	B + C	+

rTMS, repeated transcranial magnetic stimulation; A, HAMD score; B, IL-6 level; C, TNF-α level; D, NIHSS score; E, MMSE score; F, effective rate.

### Quality assessment

We evaluated the quality of the included studies according to the PEDro quality assessment scale (Table 2), and most of the included studies had methodological deficiencies in the blinding of subjects, therapists, and assessors. Three studies scored as high quality (eight scores), and 13 were scored as high quality (six scores).

**Table 2:** Evaluation of the quality of the included documents through PEDro.

**Study**	**1**	**2**	**3**	**4**	**5**	**6**	**7**	**8**	**9**	**10**	**11**	**Total score**	**Level**
Li et al. (33)	**√**	**√**	**×**	**√**	**×**	**×**	**×**	**√**	**√**	**√**	**√**	6	Medium
Jia et al. (32)	**√**	**√**	**×**	**√**	**×**	**×**	**×**	**√**	**√**	**√**	**√**	6	Medium
Fan (31)	**√**	**√**	**×**	**√**	**√**	**√**	**×**	**√**	**√**	**√**	**√**	8	High
Yunnan et al. (36)	**√**	**√**	**×**	**√**	**×**	**×**	**×**	**√**	**√**	**√**	**√**	6	Medium
Li (34)	**√**	**√**	**×**	**√**	**×**	**×**	**×**	**√**	**√**	**√**	**√**	6	Medium
Kejiao (38)	**√**	**√**	**×**	**√**	**×**	**×**	**×**	**√**	**√**	**√**	**√**	6	Medium
Leyu and Liju (29)	**√**	**√**	**×**	**√**	**×**	**×**	**×**	**√**	**√**	**√**	**√**	6	Medium
Xiaoru et al. (37)	**√**	**√**	**×**	**√**	**×**	**×**	**×**	**√**	**√**	**√**	**√**	6	Medium
Tan et al. (35)	**√**	**√**	**√**	**√**	**√**	**×**	**×**	**√**	**√**	**√**	**√**	8	High
Wang et al. (27)	**√**	**√**	**×**	**√**	**×**	**×**	**×**	**√**	**√**	**√**	**√**	6	Medium
Wang et al. (25)	**√**	**√**	**×**	**√**	**×**	**×**	**×**	**√**	**√**	**√**	**√**	6	Medium
Jie (23)	**√**	**√**	**×**	**√**	**×**	**×**	**×**	**√**	**√**	**√**	**√**	6	Medium
Niu and Wu (24)	**√**	**√**	**×**	**√**	**×**	**×**	**×**	**√**	**√**	**√**	**√**	6	Medium
Guobing (26)	**√**	**√**	**×**	**√**	**×**	**×**	**×**	**√**	**√**	**√**	**√**	6	Medium
Tian and Jia (30)	**√**	**√**	**×**	**√**	**×**	**×**	**×**	**√**	**√**	**√**	**√**	6	Medium
Liu et al. (2)	**√**	**√**	**√**	**√**	**√**	**×**	**×**	**√**	**√**	**√**	**√**	8	High

1 = inclusion exclusion criteria; 2 = randomized group; 3 = allocation concealment; 4 = similar baseline; 5 = subject blinding; 6 = therapist blinding; 7 = assessor blinding; 8 = more than 85% of patient measures; 9 = intention to treat; 10 = between-group analysis; 11 = at least one point measure (**√**: yes, no risk; **×**: no, risky).

### Meta-analysis results

#### Primary outcomes

##### Result of the HAMD score

**Figure 3:**
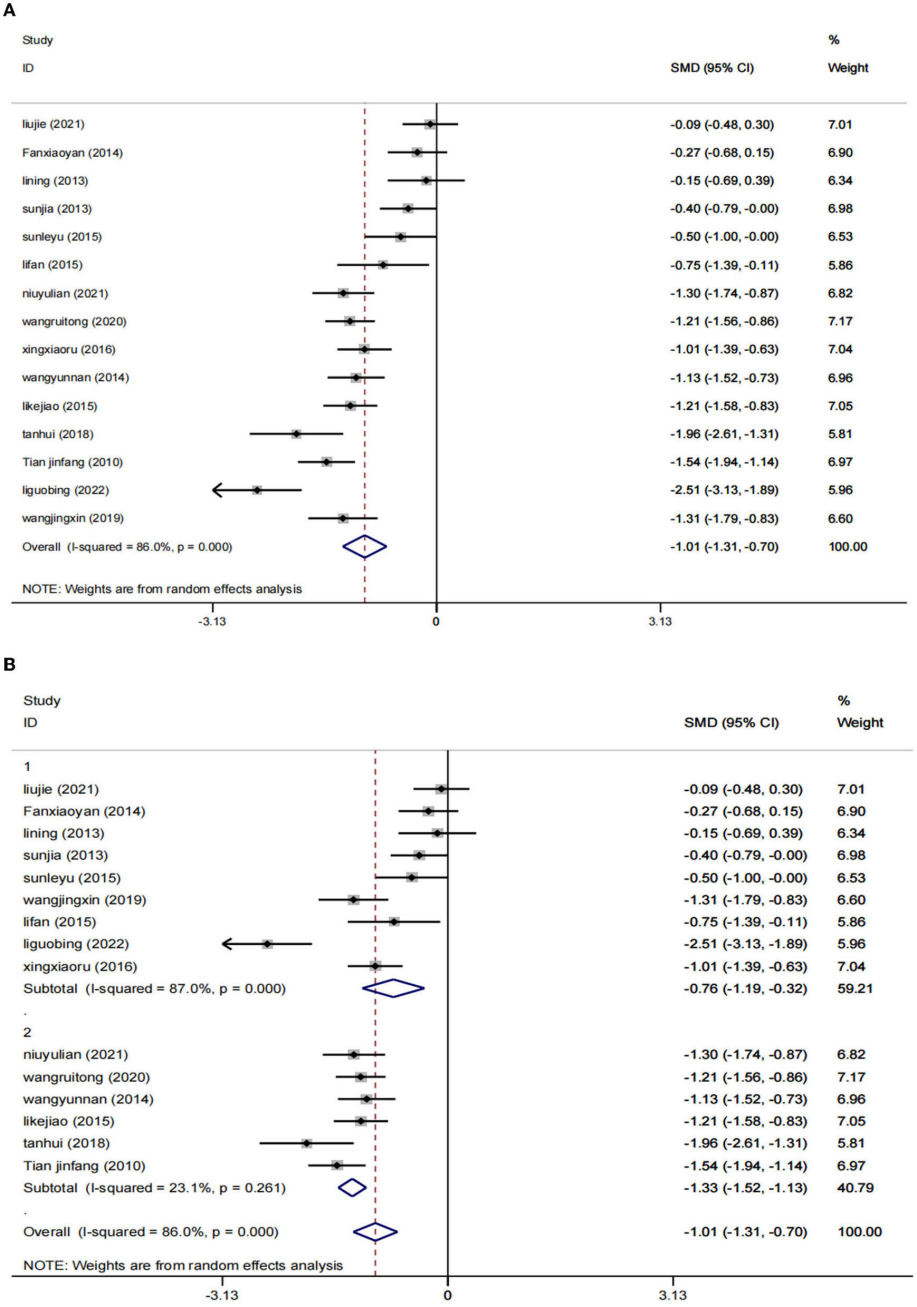
Results of HAMD score. **(A)** All studies. **(B)** After subgroup analysis (1: ≤4 weeks; 2: ≥8 weeks).

A total of 15 RCTs (23–27, 29–38), including 1,393 patients, reported HAMD scores. The HAMD scores of 15 studies were analyzed, showing statistical heterogeneity among the studies (*I*^2^ = 86.0%, *p* = 0.000). The random effect model was used for meta-analysis. The results showed that the treatment effect of the intervention group was better than that of the control group [SMD = −1.01, 95% CI (−1.31, −0.70); Figure 3A], which could prove that low-frequency rTMS combined with antidepressants had a positive effect on the improvement of depression in PSD patients.

The studies were stratified according to the intervention period of ≥8 weeks (six RCTs) or ≤4 weeks (nine RCTs). The results showed that a total of 9 RCTs (23, 26, 27, 29, 31–34, 37) were included in the subgroup of the intervention period ≤4 weeks, including 721 patients. Compared to the control group [SMD = −0.76, 95% CI (−1.19, −0.32), *I*^2^ = 87%, *p* = 0.001 < 0.05], the combination of low-frequency rTMS and antidepressants was more effective in improving the symptoms of patients with PSD. In the subgroup of the intervention period of ≥8 weeks, six RCTs (24, 25, 30, 35, 36, 38) were included, totaling 672 patients. The results were statistically significant compared to the control group [SMD = −1.33, 95% CI (−1.31, −0.70), *p* = 0.000 < 0.05] by the random effects model (*I*^2^ = 23.1%). This proves that low-frequency rTMS combined with antidepressants has positive significance in improving patients' symptoms. Furthermore, we can speculate from the results that the duration of the intervention ≥8 weeks may be more effective (Figure 3B).

##### Effective rate

**Figure 4:**
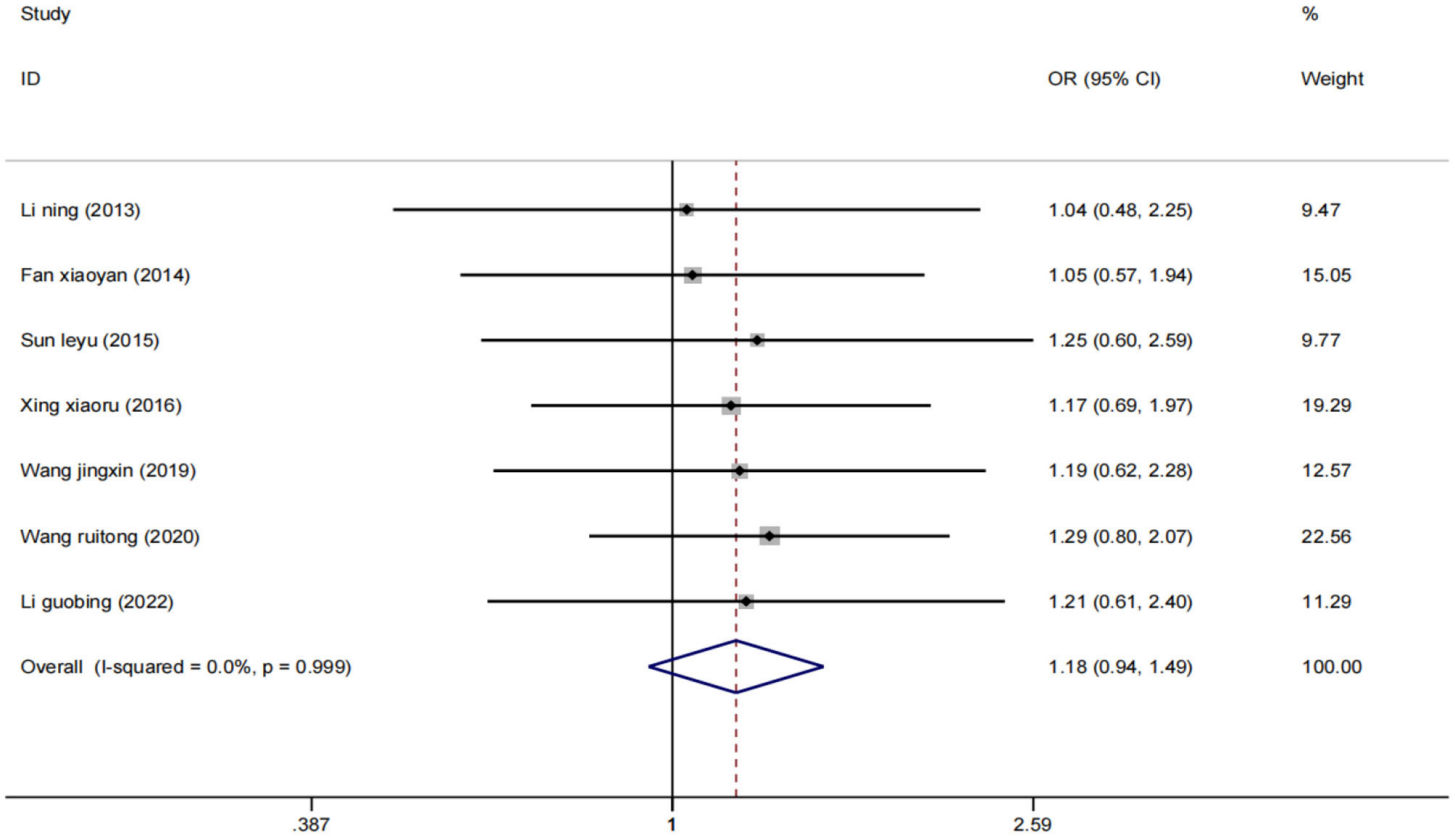
Forest plot of total effective rate.

Seven studies (25–27, 29, 31, 33, 37) evaluated 631 participants and reported overall response rates. Data were pooled using a fixed effect model (*I*^2^ = 0%) (Figure 4), and the results showed that low-frequency rTMS combined with antidepressants was adequate for the treatment of PSD compared with the control group [OR = 1.18, 95% CI (0.94, 1.49), *p* = 0.999].

#### The secondary outcomes

##### Serum IL-6 and TNF-α levels

**Figure 5:**
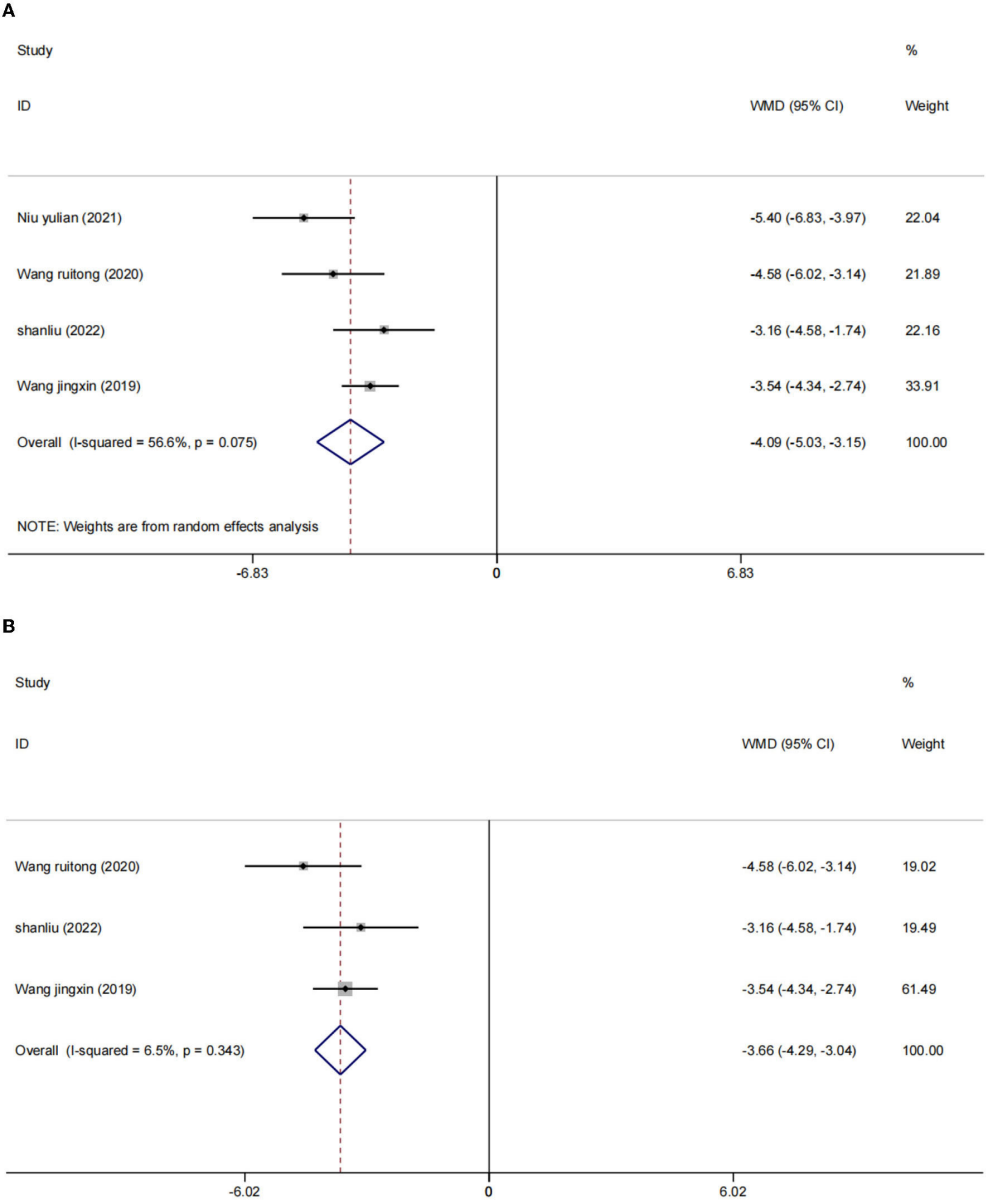
Forest plot of IL-6 level in serum. **(A)** All studies. **(B)** After sensitivity analysis.

**Figure 6:**
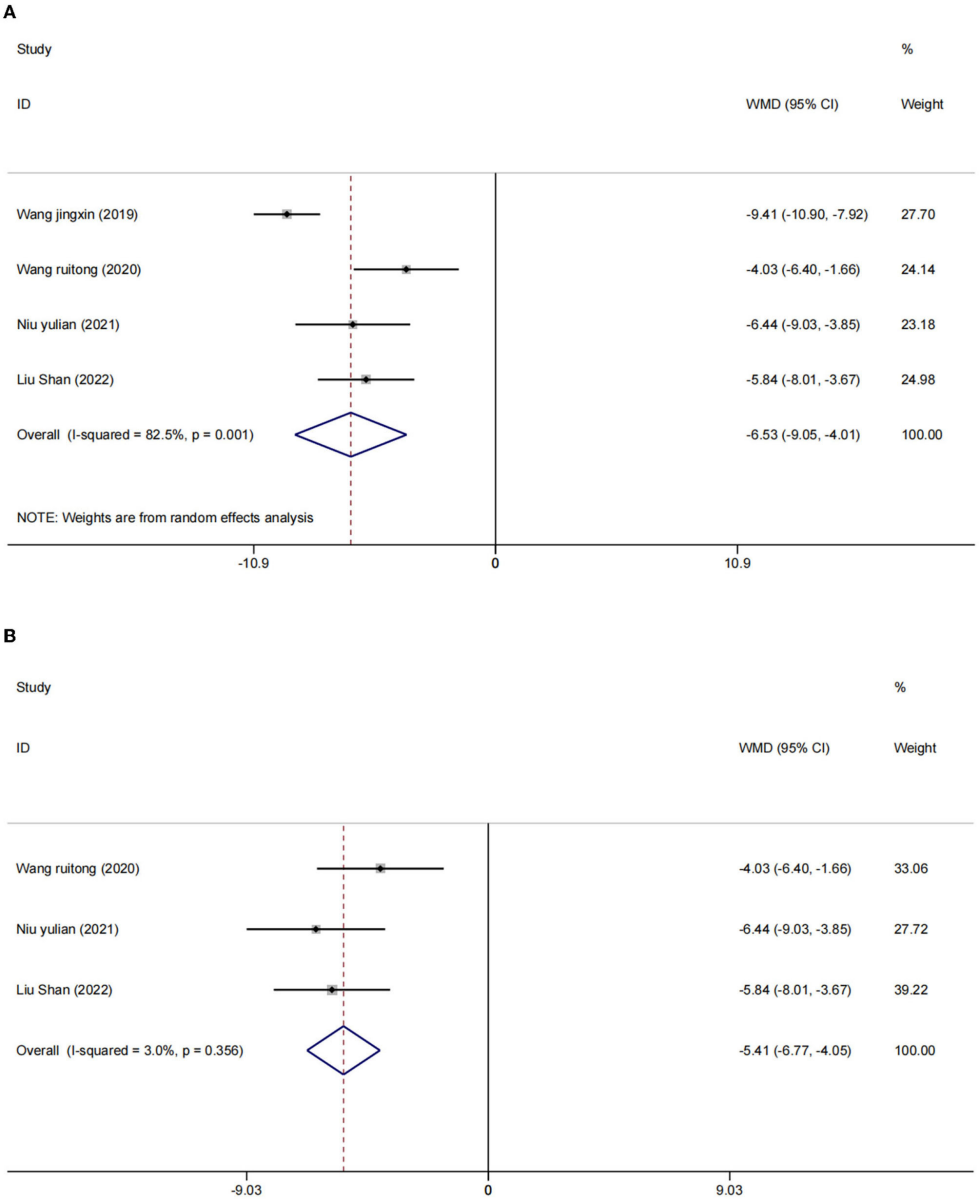
Forest plot of TNF-α level in serum. **(A)** All studies. **(B)** After sensitivity analysis.

There were four studies (2, 24, 25, 27) in which the blood inflammatory factors interleukin-6 (IL-6) and tumor necrosis factor-α (TNF-α) were measured in 402 participants. We used a random effects model to combine the data. The results showed that compared to the control group, the intervention group had a significant reduction in IL-6 [WMD = −4.09, 95% CI (−5.03, −3.15), *I*^2^ = 56.6%, *p* = 0.075] (Figure 5A) and TNF-α [*I*^2^ = 82.5%, *p* = 0.001], WMD = −6.53, 95% CI (−9.05, −4.01; Figure 6A) were more effective.

After sensitivity analysis, we found that the heterogeneity decreases after the exclusion of two studies (24, 27). We used the fixed effect model [IL-6 (WMD = −3.66, 95% CI (−4.29, −3.04), *I*^2^ = 6.5%, *p* = 0.075); Figure 5B] and [TNF-α (WMD = −5.41, 95% CI (−6.77, −4.05), *I*^2^ = 3.0%, *p* = 356); Figure 6B]. We considered that the heterogeneity might be due to the different stimulation sites and the intervention period (left frontal lobe, 4 weeks) in this study compared with the other two studies (right frontal lobe, 8 weeks).

##### NIHSS score

**Figure 7:**
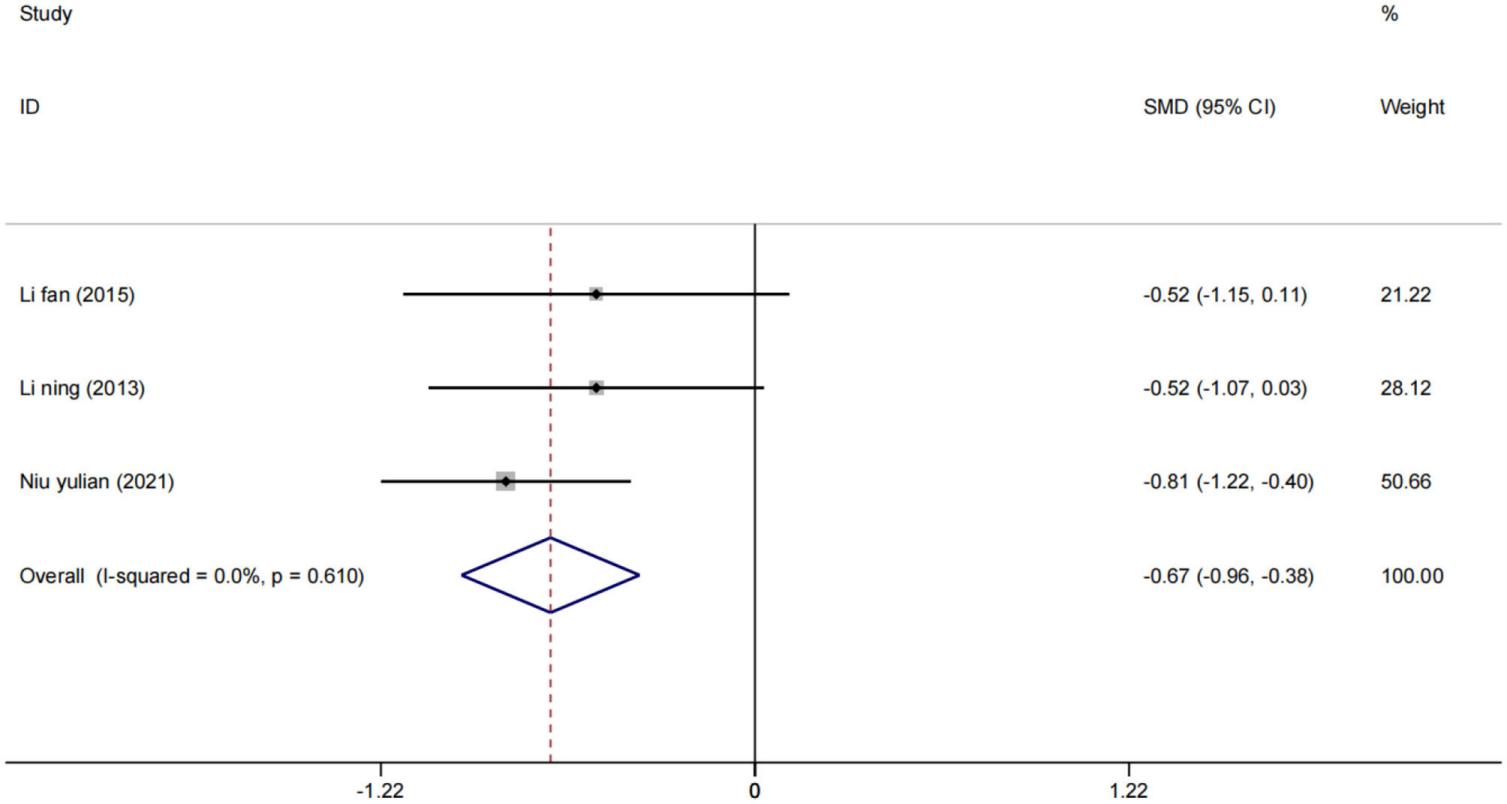
Forest plot of the NIHSS score.

A total of three studies (24, 33, 34) assessed NIHSS scores in 193 patients. Using a fixed effect model, the statistical significance was found compared to the control group [SMD = −0.67, 95% CI (−0.96, −0.38), (*I*^2^ = 0%), *p* = 0.610; Figure 7], which showed that low-frequency rTMS combined with antidepressants also helped to improve neurological function in stroke patients.

##### MMSE score

**Figure 8:**
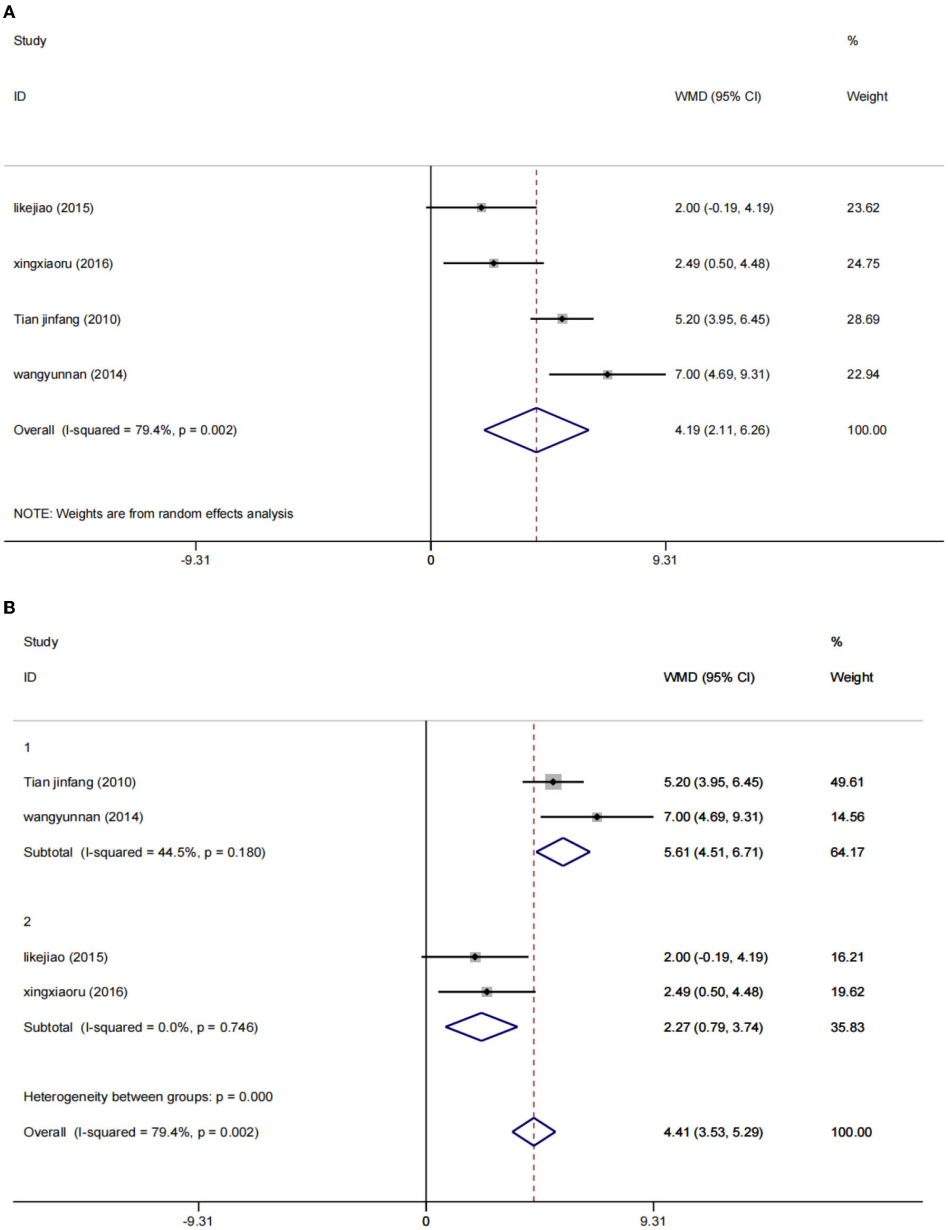
Result of MMSE score. **(A)** All studies. **(B)** After subgroup analysis (1: 0.5 HZ; 2: 1 HZ).

A random effect model was used to perform a meta-analysis of the MMSE scores of 486 participants included in four studies (30, 36–38). The increase of MMSE score in the low-rTMS combined with the antidepressant group was significantly higher than that in the control group [WMD = 4.19, 95% CI (2.11, 6.26), *I*^2^ = 79.4%, *p* = 0.002] (Figure 8A).

Due to the significant heterogeneity (*I*^2^ = 79.4%), the intervention frequency (NOTE. 1: 0.5 Hz; 2: 1 Hz) performed a subgroup stratified analysis, reducing the heterogeneity to a reasonable range. Using the fixed effects model, the results show that WMD = 5.61, 95% CI (4.51, 6.71), *I*^2^ = 44.5%, *p* = 0.180 and WMD = 2.27, 95% CI (0.79, 3.74), *I*^2^ = 0%, *p* = 0.746. Low rTMS combined with antidepressants can significantly improve the MMSE score of PSD patients, which can improve the cognitive function of PSD patients (Figure 8B).

#### Sensitivity analysis

**Figure 9:**
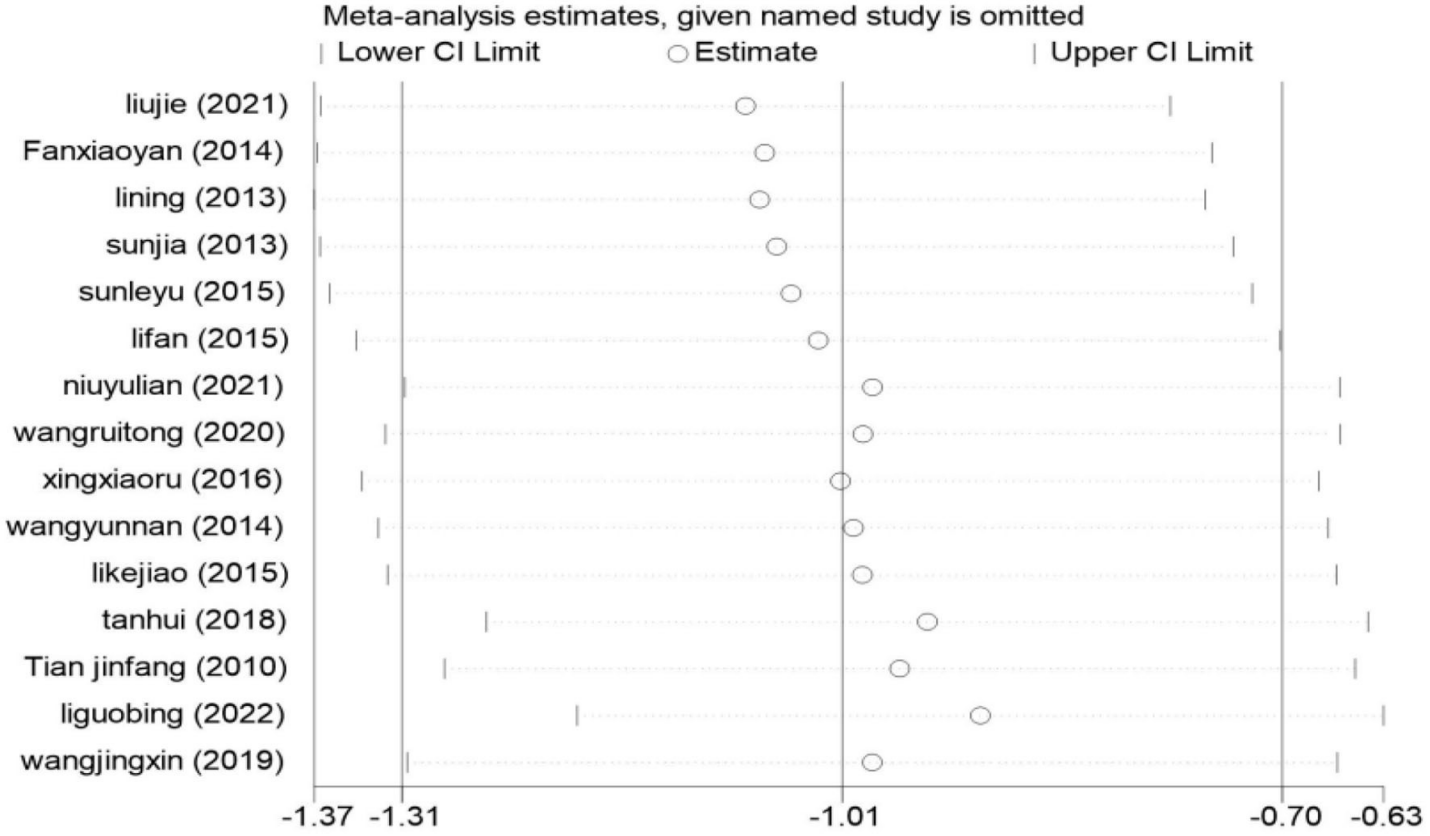
Result of sensitivity analysis.

We used StataMP 17 for sensitivity analysis on the result of the HAMD score; the results are shown in Figure 9. There were 15 studies included in the meta-analysis, and the pooled results of the remaining studies were not statistically significant regardless of which one was excluded. The results were consistent with the original combined results [SMD = −1.01, 95% CI (−1.31, −0.70)], which proved that the meta-results were stable.

#### Meta-regression results

**Figure 10:**
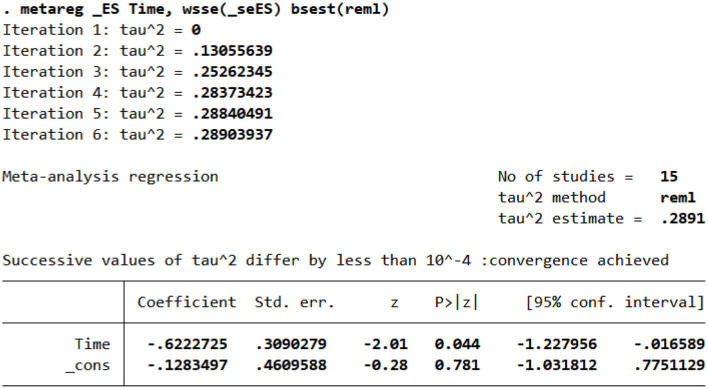
Meta-regression results of the intervention period.

Since the combined results of HAMD score data showed significant heterogeneity (*I*^2^ = 86.0% > 50%), we conducted meta-regression according to the intervention frequency, site, and intervention period of the included literature to find the source of heterogeneity. The results showed that the intervention period (*p* = 0.044 < 0.05) was the source of heterogeneity (Figure 10). Neither the frequency of intervention (*p* = 0.636 > 0.05) nor the site of intervention (*p* = 0.542 > 0.05) was the source of heterogeneity.

#### Risk of bias

**Figure 11:**
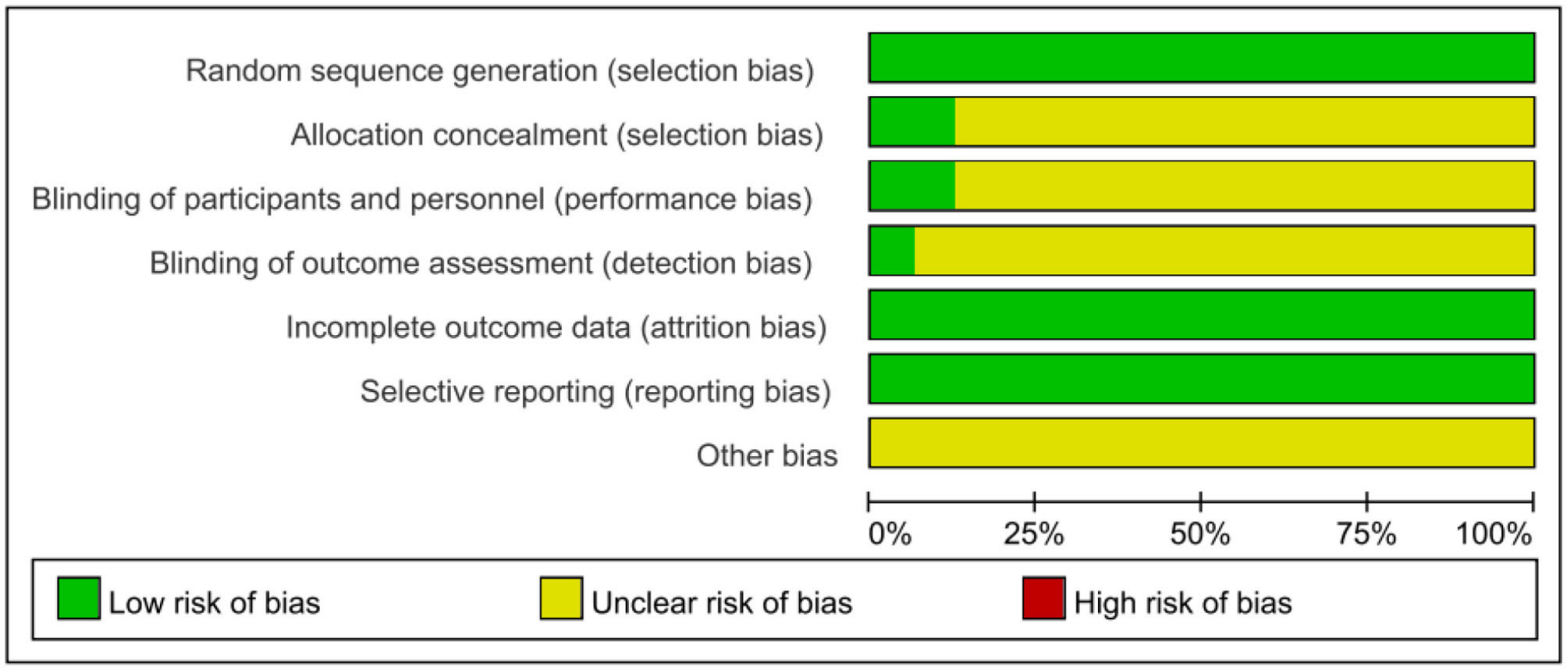
Risk of bias graph.

**Figure 12:**
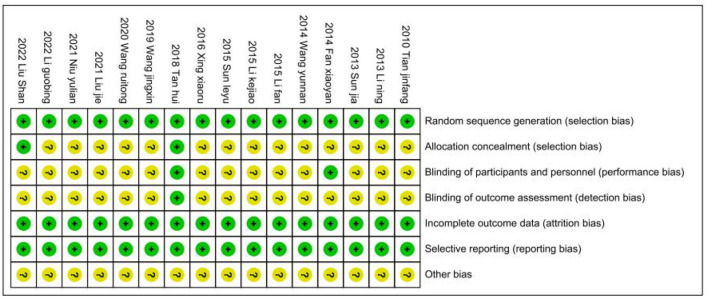
Risk of bias summary.

We followed the Cochrane Handbook for Systematic Reviews of Interventions 5.3 to evaluate the risk of bias, and two researchers (JHP and YW) independently evaluated the risk of bias in the included studies. After integrating the results, the 16 included RCTs were shown to be at low risk (Figures 11, 12). Because we could not obtain the original study protocols of the included kinds of literature, other biases could not be determined and were evaluated as unknown risks after discussion by three researchers (JHP, YW, and YSW).

#### Publication bias

**Figure 13:**
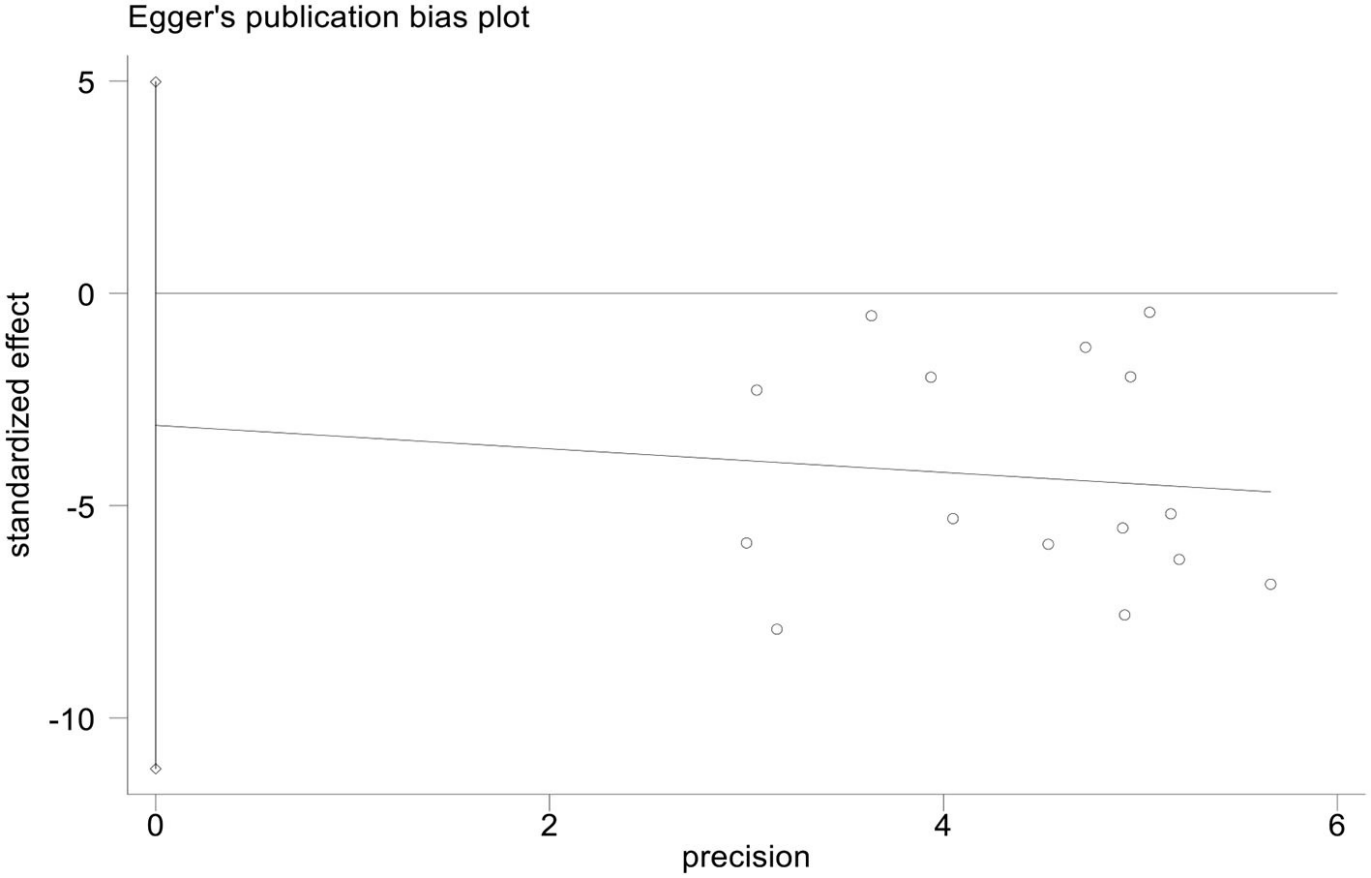
Egger's publication bias plot of HAMD scores.

**Figure 14:**
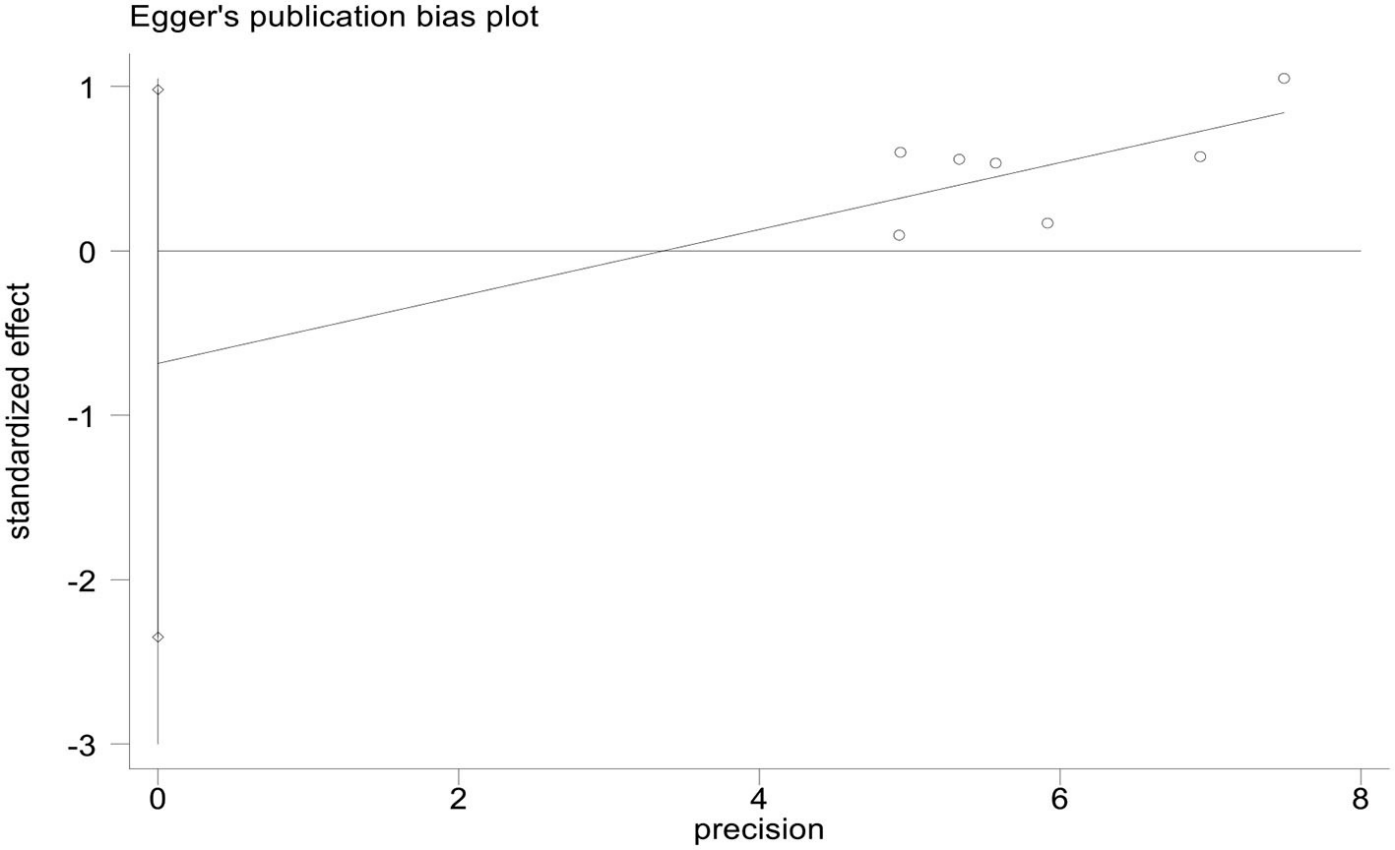
Egger's publication bias plot of effective rate.

We used StataMP 17 to analyze the publication bias of the HAMD score and total effective rate by Egger's test (2). The results showed that the Egger's test of the HAMD score and the total effective rate was *p* = 0.421 > 0.05 and *p* = 0.339 > 0.05, showing no significant publication bias (Figures 13, 14).

## Discussion

This meta-analysis included 16 studies with 1,463 patients with PSD. The experimental group used rTMS combined with antidepressants (such as fluoxetine, duloxetine, mirtazapine, paroxetine, or flupentixol melitracen), and the control group received antidepressants (Table 1). We used PEDro to assess the quality of the included studies, including two high-quality studies (eight scores) and 13 medium-quality studies (six scores). The risk of bias was evaluated as low because the 16 studies described the randomization method and reported the primary outcome measures. However, we did not have access to the original protocol and other risks of bias evaluations are unclear. We used Egger's test to analyze the publication bias of the HAMD score and the total response rate, and the results did not show a significant publication bias of the HAMD score and the total response rate (*p* = 0.421 > 0.05 and *p* = 0.339 > 0.05).

The results of the meta-analysis proved that rTMS combined with antidepressants could reduce the HAMD score of PSD patients compared to an antidepressant [SMD = −1.01, 95% CI (−1.31, −0.70), *I*^2^ = 86.0%, *p* = 0.000]. However, there was significant heterogeneity. In order to find the source of heterogeneity, we conducted a meta-regression according to publication year, age, intervention frequency, intervention period, intervention site, and intervention drug. We also performed subgroup analyses according to the intervention period (≤4 and ≥8 weeks). The results show that SMD = 0.76, 95% CI (1.19, 0.32), *I*^2^ = 87%, *p* = 0.001 < 0.05 (4 weeks) or less, and SMD = 1.33, 95% CI (1.31, 0.70), *I*^2^ = 23.1%, *p* = 0.000 < 0.05 (≥8 weeks). Low rTMS combined with an antidepressant had a positive effect in improving the depression of PSD patients. In order to further verify the stability of the meta-results, we conducted a sensitivity analysis. The results showed that no matter which study was excluded, the combined results of the remaining studies were not statistically significant, consistent with the original combined results [SMD = −1.01, 95% CI (−1.31, −0.70)], and the results were stable.

Previous studies have shown that the pathological mechanism of PSD may be related to the inflammatory process caused by stroke, and serum levels of interleukin-1β (IL-1β), interleukin-6 (IL-6), IL-10, interferon-γ, and the TNF-α level in PSD patients increased to varying degrees (40–42). Furthermore, it will increase with an increase in the degree of depression. The results of this meta-analysis evidenced that rTMS combined with antidepressants could reduce the levels of serum inflammatory factors (IL-6 [WMD = −3.66, 95% CI (−4.29, −3.04), *I*^2^ = 6.5%, *p* = 0.075]) and TNF-α [WMD = −5.41, 95% CI (−6.77, −4.05), *I*^2^ = 3.0%, *p* = 356]). It could be seen that low-frequency repetitive transcranial magnetic stimulation could increase the protective factors of nerve cells. In contrast, the expression of factors IL-6 and TNF-α that damage nerve cells decreases. Therefore, there is evidence that low-frequency repetitive transcranial magnetic stimulation could somewhat protect nerve cells by inhibiting the production of inflammatory factors related to depression (43–45).

This study also demonstrated that low rTMS combined with antidepressants could reduce NIHSS scores [SMD = −0.67, 95% CI (−0.96, −0.38), *p* = 0.610] and increase the MMSE score [WMD = 4.19, 95% CI (2.11, 6.26)] in PSD patients. There is promising data that the low-rTMS combined with antidepressants possibly improve neurological function in stroke patients and the cognitive function of PSD patients to some extent.

Currently, research on the mechanism of PSD is not very detailed and most of the clinical treatments are symptomatic. However, some studies have also shown that the appearance of PSD may be related to the following factors. First, dysfunction of the central monoamine neurotransmitter system. The experiments of Whyte et al. (46) showed that the destruction of noradrenergic and serotonergic neurons led to varying degrees of decrease in norepinephrine and serotonin after PSD, which can lead to the occurrence of depression. Relevant experiments by Robinson et al. (47) also showed that the levels of 5-HT, NE, and DA neurotransmitters in the hippocampus and frontal cortex of PSD rats were significantly reduced, leading to the appearance of depression.

Meanwhile, the significant efficacy of SSRI and SNRI in treating PSD has confirmed this speculation. Second, the appearance of oxidative stress. Nabavi et al. (48) believed that reactive oxygen species produced in the stroke process could cause oxidative stress, lipid peroxidation, protein oxidation, and DNA damage in the nerve tissue, which induced post-stroke depression. The study of Nabnvi et al. (49) also provided favorable evidence for the hypothesis. Third, a decrease in neurotrophic factors. Xia et al. (50) showed that the occurrence of PSD negatively correlated with the level of neurotrophic factor (BDNF). The study of Moghbelinejad et al. (51) also proved that BDNF could effectively regulate the regeneration and apoptosis of neurons, mediate the growth and proliferation of nerve cells, and protect nerve tissues from the damage caused by neuronal death caused by ischemia or depression. Fourth, psychosocial changes. Brggimann et al. (52) showed that posttraumatic stress disorder was associated with changes in psychological status after stroke. Moreover, there are also relevant studies (53, 54) that the degree of social support at 3–6 months, 1 year, and 2 years after stroke is related to the severity of PSD.

A total of five studies (24, 26, 32, 34, 38) reported adverse effects, mainly including neurological and digestive abnormalities. Neurological abnormalities mainly included scalp discomfort, sweating, drowsiness, fatigue, and headache. Digestive abnormalities include nausea, vomiting, loss of appetite, and constipation. In the low-frequency rTMS combined with the antidepressant group, scalp discomfort occurred in three cases, headache in 20 cases (14 cases relieved after rest and six cases relieved after taking acetaminophen), fatigue in two cases, nausea in two cases, and vomiting in one case. In the antidepressant group, there were three cases of drowsiness, two cases of headache, four cases of constipation and loss of appetite, five cases of nausea, and five cases of vomiting. These adverse effects may be related to the administration of antidepressants.

This study also has some limitations. First, as shown in Table 2 and Figures 11, 12, most of the studies included in this study had methodological deficiencies and mostly did not blind participants, therapists, and assessors. These factors may affect the effect of low rTMS combined with antidepressants in the treatment of PSD, so more high-quality and large-sample studies are needed to confirm it in the future. Second, most of the intervention periods of the 16 included studies were between 4 weeks and 8 weeks, and there were no reports on long-term follow-up, thus, the long-term efficacy of these studies could not be analyzed, which may be an issue we should consider in the future. Third, only three studies were recovered according to the inclusion criteria and used the inflammatory factors IL-6 and TNF-α as outcome indicators. Although our conclusion is positive and supported by research, it is still necessary to verify this conclusion with more high-quality studies in the future. Finally, as shown in Table 1, there were differences in intervention sites and intervention frequencies in the 16 included studies. Unfortunately, in this meta-analysis, we only compared the efficacy of low-frequency rTMS and antidepressants and did not compare the efficacy between different sites. A new meta-analysis can be carried out in this regard in the future.

## Conclusion

The results of this meta-analysis evidenced the efficacy and safety of low-rTMS combined with antidepressants in the treatment of depression in PSD patients. The combined therapy could reduce The depression state and the levels of IL-6 and TNF-α, and enhance the cognitive function of patients. In addition, low-rTMS had fewer adverse effects, proving safety. However, there are shortcomings, such as a lack of long-term follow-up, different intervention sites of low-rTMS, and different intervention frequencies (0.5 or 1 Hz). In the future, more large sample size, higher quality, and more extended observations of RCTs are needed to verify further effectiveness of low-rTMS combined therapy on PSD. A new meta-analysis could determine which intervention sites and frequency are more effective in treating PSD.

## Data availability statement

The original contributions presented in the study are included in the article/supplementary material, further inquiries can be directed to the corresponding authors.

## Author contributions

JP, DZ, YW, LL, HL, YW, and SJ contributed equally to this meta-analysis. JP, DZ, and SJ conceived the study and drafted the first framework of the manuscript. JP, YW, LL, HL, and YW were responsible for data collection and verification. JP and DZ contributed significantly to the revision of the study. All authors contributed to the design, information gathering, data collection, analysis, writing, and final editing.
